# Efficacy and safety of cefazolin versus antistaphylococcal penicillins for the treatment of methicillin-susceptible *Staphylococcus aureus* bacteremia: a systematic review and meta-analysis

**DOI:** 10.1186/s12879-018-3418-9

**Published:** 2018-10-11

**Authors:** Changcheng Shi, Yubo Xiao, Qi Zhang, Qingyu Li, Fei Wang, Jing Wu, Nengming Lin

**Affiliations:** 1 Department of Clinical Pharmacy, Affiliated Hangzhou First People’s Hospital, Zhejiang University School of Medicine, Hangzhou, China; 2 Department of Clinical Pharmacy, Hangzhou First People’s Hospital, Nanjing Medical University, Hangzhou, China; 3 Department of Pharmacometrics, Mosim Co., Ltd, Shanghai, China; 4 Department of Pharmacy, Hangzhou Obstetrics & Gynecology Hospital, Hangzhou, China; 5 Department of Clinical Pharmacology, Translational Medicine Research Center, Affiliated Hangzhou First People’s Hospital, Zhejiang University School of Medicine, Hangzhou, China

**Keywords:** Cefazolin, Antistaphylococcal penicillins, Methicillin-susceptible *Staphylococcus aureus*, Bacteremia, Meta-analysis

## Abstract

**Background:**

Antistaphylococcal penicillins (ASPs) and cefazolin have become the most frequent choices for the treatment of methicillin-susceptible *Staphylococcus aureus* (MSSA) infections. However, the best therapeutic agent to treat MSSA bacteremia remains to be established.

**Methods:**

We conducted a systematic review and meta-analysis to evaluate the efficacy and safety of these two regimens for the treatment of MSSA bacteremia. PubMed, EMBASE and the Cochrane Library from inception to February 2018 were searched. The primary outcome was mortality. The secondary outcomes included treatment failure, recurrence of bacteremia, adverse effects (AEs) and discontinuation due to AEs. Data were extracted and pooled odds ratios (ORs) and 95% confidence intervals (CIs) were calculated.

**Results:**

A total of ten observational studies met the inclusion criteria. The results indicate that compared to ASPs, cefazolin was associated with significant reduction in mortality (OR, 0.69; 95% CI, 0.58 to 0.82; I^2^ = 3.4%) and clinical failure (OR, 0.56; 95% CI, 0.37 to 0.85; I^2^ = 44.9%) without increasing the recurrence of bacteremia (OR, 1.12; 95% CI, 0.94 to 1.34; I^2^ = 0%). There were no significant differences for the risk of anaphylaxis (OR, 0.91; 95% CI, 0.36 to 2.99; I^2^ = 0%) or hematotoxicity (OR, 0.56; 95% CI, 0.17 to 1.88; I^2^ = 0%). However, nephrotoxicity (OR, 0.36; 95% CI, 0.16 to 0.81; I^2^ = 0%) and hepatotoxicity (OR, 0.12; 95% CI, 0.04 to 0.41; I^2^ = 0%) were significantly lower in the cefazolin group. Moreover, cefazolin was associated with lower probability of discontinuation due to AEs compared with the ASPs (OR, 0.24; 95% CI, 0.12 to 0.48; I^2^ = 18%).

**Conclusion:**

The results of present study favor the application of cefazolin and should be regarded as important evidence to help make clinical decisions in choosing a treatment option for treating MSSA bacteremia.

**Electronic supplementary material:**

The online version of this article (10.1186/s12879-018-3418-9) contains supplementary material, which is available to authorized users.

## Background

*Staphylococcus aureus* is the principal cause of community-acquired and nosocomial infections. Although more clinical research has focused on the methicillin-resistant *Staphylococcus aureus* (MRSA), bloodstream infections due to methicillin-susceptible *Staphylococcus aureus* (MSSA) remain a significant healthcare burden worldwide with high morbidity and mortality [1, 2].

Prevailing evidence has established the use of β-lactam antibiotics in preference to vancomycin as optimum therapy for MSSA bacteremia [3, 4]. According to the relevant clinical practice guidelines, cefazolin and antistaphylococcal penicillins (ASPs) such as nafcillin, oxacillin, and cloxacillin are the most frequent choices [5–8]. However, the optimal choice of β-lactam antibiotic for MSSA bacteremia is still unclear. To our knowledge, randomized control trials (RCTs) directly comparing clinical outcomes between cefazolin and ASPs for MSSA bacteremia are lacking, but data are emerging to support the role of cefazolin as a first-line agent for MSSA bacteremia [9, 10]. The aim of this meta-analysis was to summarize all the available evidence and compare the efficacy and safety of cefazolin versus ASPs for the treatment of MSSA bacteremia.

## Methods

### Registry

This systematic review and meta-analysis has been prospectively registered in PROSPERO with the registration no. CRD42018090547.

### Literature search strategy

We searched PubMed, EMBASE and the Cochrane Library from inception to February 2018. The search terms were “(oxacillin OR nafcillin OR methicillin OR cloxacillin OR floxacillin OR dicloxacillin OR flucloxacillin OR antistaphylococcal penicillin OR semisynthetic penicillin) AND (methicillin-susceptible *Staphylococcus aureus* OR methicillin susceptible *Staphylococcus aureus* OR MSSA) AND (bacteremia OR bacteraemia OR bloodstream infection OR sepsis) AND cefazolin)”. Reference lists of the relevant publications were searched for additional literature. No language or publication restriction were imposed.

### Inclusion criteria and study selection

Two review authors independently reviewed the results to screen relevant studies for further assessment. Any discrepancy was resolved through discussion. Original studies included in our meta-analysis were required to meet the following criteria: (i) RCTs or observational designs, including cohort and case-control studies; (ii) comparing the efficacy or safety of treatment between cefazolin and ASPs for MSSA bacteremia in two groups of patients; (iii) at least one of the following outcomes: mortality, treatment failure, recurrence of bacteremia, adverse effects (AEs) and discontinuation due to AEs. Exclusion criteria were as follows: (i) no outcome data were available; (ii) studies investigating only cefazolin or ASPs; (iii) presented solely as abstract at scientific conferences; (iv) duplicate publications.

### Outcomes

The primary outcome assessed in this meta-analysis was mortality. The secondary outcomes included treatment failure, recurrence of bacteremia, AEs and discontinued treatment due to AEs. While extracting data, we found that for ‘mortality’, some studies reported ‘all-cause mortality’, ‘all-cause in-hospital mortality’, ‘over mortality’ or ‘bacteremia-associated mortality’. In our meta-analysis, all of these terms were regarded as approximate ‘mortality’. When data for more than one endpoint were available, mortality in the main analysis was recorded at the latest point in the study (e.g., 90-day mortality had precedence over 30-day mortality). Treatment failure and other secondary outcomes were defined according to descriptions provided by each study.

### Data extraction and quality assessment

The variables that were abstracted from each study included the investigator, publication year, study design, location, enrolment period, number of participants, patient characteristics (age, sex, disease severity, the source of infection), intervention and comparison, dose and duration of administration, and outcome measures. Data were extracted independently by two authors. Discrepancies were resolved in meetings. Because only observational studies were available for inclusion, the quality of the included publications was appraised based on the Newcastle-Ottawa scale (NOS), as recommended by the Cochrane Collaboration [11]. Each study was scored from 0 to 9, based on eight items within the three following domains: selection, comparability and exposure (or outcome) [12].

### Statistical analysis

Pooled odds ratios (ORs) and 95% confidence intervals (CIs) of all outcomes were used to determine whether there were significant differences between the compared data. Heterogeneity was evaluated using the I^2^ statistic and a value of > 50% was defined to indicate significant heterogeneity. If no significant heterogeneity was found, a fixed-effects model was used. Otherwise, a random-effect model was selected. Sensitivity analyses were undertaken by excluding each publication. Subgroup analyses were performed based on the study design, location, study period, time of mortality reporting and control group. Additionally, we presented separate subgroup analyses of adjusted or unadjusted estimates. Publication bias was assessed by generating funnel plots and tested used Begg’s and Egger’s asymmetry test. A *P*-value of < 0.05 was considered statistically significant. Meta-analysis statistical analyses were performed using STATA software (version 12.0, Stata Corporation, College Station, TX, USA).

## Results

### Search results

**Fig. 1:**
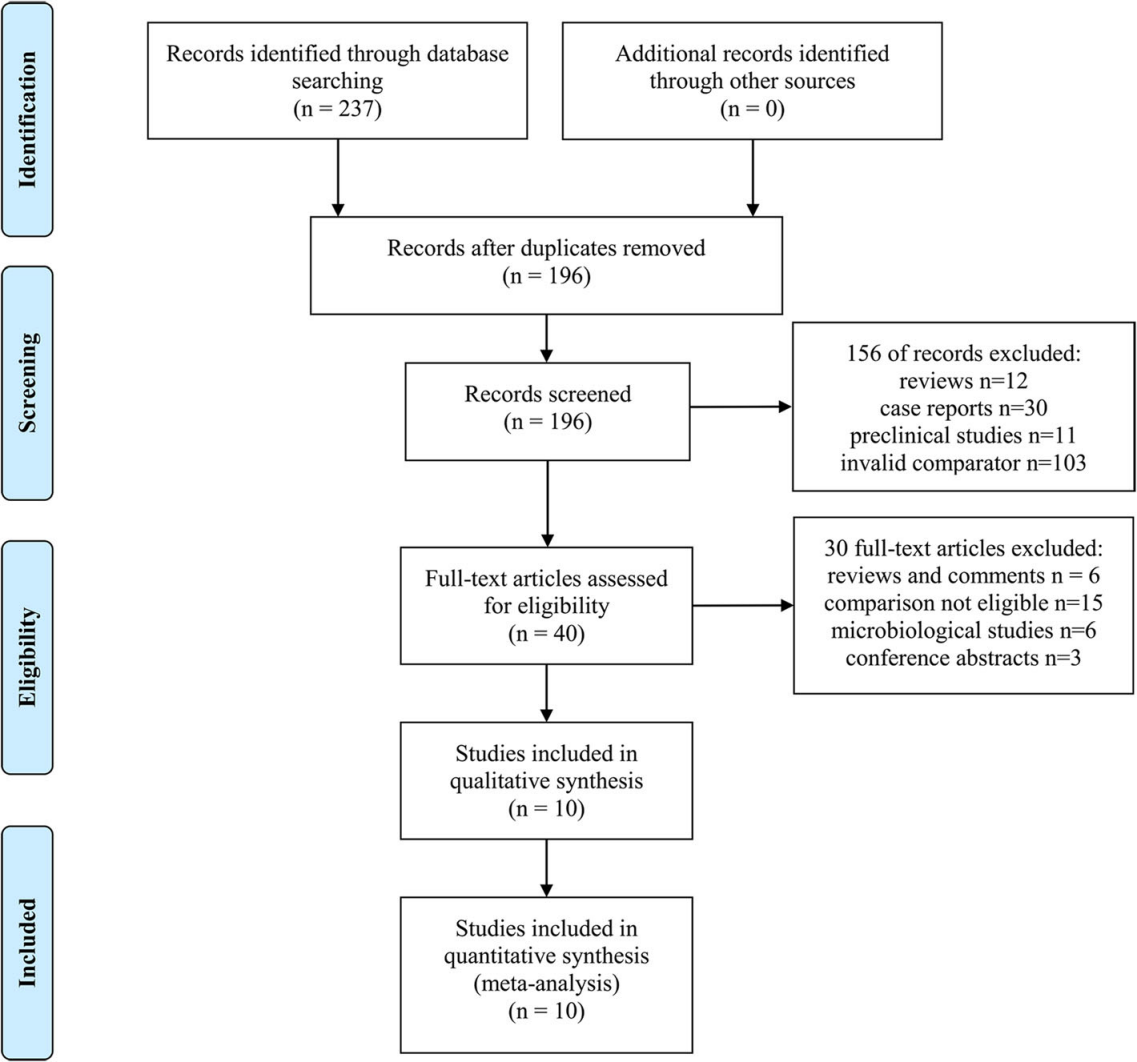
Flow diagram of the literature search and selection process

A total of 237 articles were identified in the initial search. After selection, ten studies involving 4779 patients met our inclusion criteria [13–22]. The details of the study selection process are shown in Fig. 1. Eight retrospective studies [13–18, 20, 21] and two prospective studies [19, 22] were included. No RCTs were identified. The studies were conducted in different countries as follows: the USA (5 trials) [16, 18–21], South Korea (2 trials) [13, 22], Canada (1 trial) [17], Singapore (1 trial) [15] and Israel (1 trial) [14]. The studies were published from 2011 to 2018. The mean or median patient age ranged between 50 and 69 years. Four studies compared cefazolin with nafcillin [13, 19, 20, 22], three compared cefazolin with cloxacillin [14, 15, 17], two compared cefazolin with oxacillin [16, 18], and one compared cefazolin with nafcillin or oxacillin [21]. Seven studies reported the dose or duration of administration [13, 15–20]. The detailed characteristics of included studies are represented in Table 1 and Additional file 1: Table S1. Most studies included were given five or more Newcastle-Ottawa Scale stars, indicating high quality (Additional file 1: Table S2).

**Table 1:** Characteristics of the studies included in the meta-analysis

Study	Design	Location	Study period	Treatment groups	No. of patients	Median dose (g/d)	Median duration (d)	ICU admission %	Definition of mortality
Lee 2011 [13]	SC retrospective PS-matched case- control study	South Korea	2004–2009	CFZ	49	NR	17	NR	30-day over mortality
				NAF	84	NR	15	NR	90-day SAB-related mortality
Paul 2011 [14]	SC retrospective cohort study	Israel	1988–1994 & 1999–2007	CFZ	72	NR	NR	5.2	30-day all-cause mortality
				CLX	281	NR	NR		90-day mortality
Renaud 2011 [15]	SC retrospective cohort study	Singapore	2009	CFZ	14	2	NR	NR	30-day mortality
				CLX	13	2–8	NR	NR	
Li 2014 [16]	MC retrospective cohort study	USA	2008–2012	CFZ	59	6	31	7	30-day all-cause mortality
				OXA	34	12	39	18	90-day all-cause mortality
Bai 2015 [17]	MC retrospective PS-matched cohort study	Canada	2007–2010	CFZ	105	3	NR	10	90-day mortality
				CLX	249	12	NR	18	
Rao 2015 [18]	MC retrospective cohort study	USA	2010–2013	CFZ	103	6	29	41.8	All-cause in-hospital mortality
				OXA	58	12	32.5	32.8	
Pollett 2016 [19]	SC prospective PS-matched cohort study	USA	2008–2013	CFZ	70	NR	20	13	90-day all-cause mortality
				NAF	30	NR	12	27	
Flynt 2017 [20]	MC retrospective cohort study	USA	2013–2015	CFZ	68	6	NR	NR	30-day all-cause mortality
				NAF	81	12	NR	NR	
McDanel 2017 [21]	MC retrospective cohort study	USA	2003–2010	CFZ	1163	NR	NR	15	30-day all-cause mortality
				NAF/ OXA	2004	NR	NR	19	90-day all-cause mortality
Lee 2018 [22]	MC prospective PS-matched cohort study	South Korea	2013–2015	CFZ	79	NR	NR	NR	90-day all-cause mortality
				NAF	163	NR	NR	NR	30-day all-cause mortality

*CFZ* cefazolin, *CLX* cloxacillin, *MC* multicenter, *NAF* nafcillin, *NR* not reported, *OXA* oxacillin, *PS* propensity score, *SC* single center

### Mortality

**Fig. 2:**
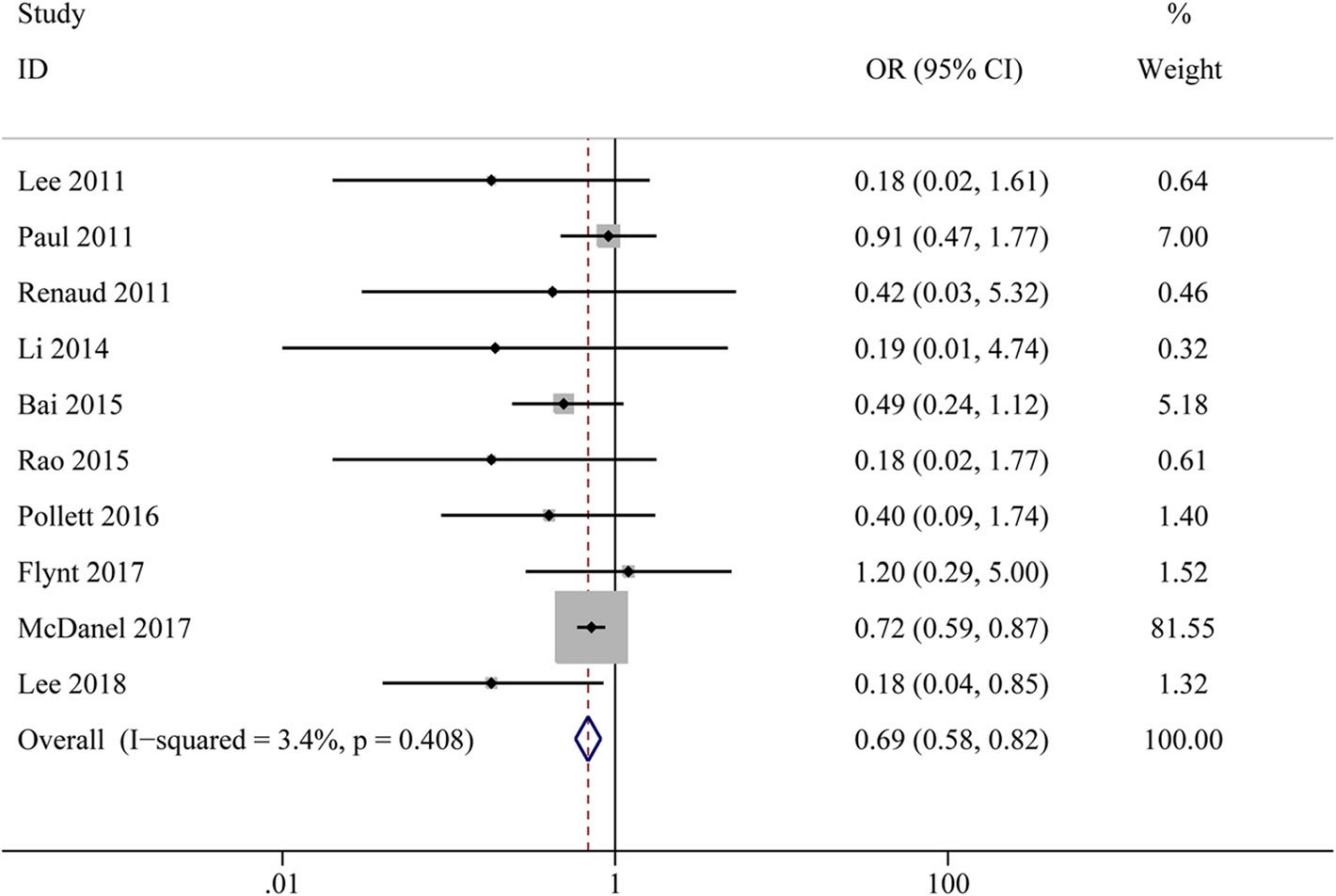
Forest plots of odds ratios for mortality

All included studies reported on mortality. Our meta-analysis indicated that mortality was significant decreased in MSSA bacteremia patients treated with cefazolin compared to those treated with ASPs (OR, 0.69; 95% CI, 0.58 to 0.82; I^2^ = 3.4%) (Fig. 2). Sensitivity analysis was used to evaluate the robustness of the findings after exclusion of each publication (Additional file 1: Table S3). We also performed subgroups analyses based on the study design, location, study period, time of mortality reporting, control group. Most of the subgroups analyses showed that cefazolin was associated with lower mortality than ASPs, except for the studies which the control group was cloxacillin. Six studies provided adjusted mortality data after controlling for potential confounders and similar results were obtained (OR, 0.69; 95% CI, 0.58 to 0.83; I^2^ = 23.6%) (Table 2).

**Table 2:** Subgroup analysis of mortality with cefazolin versus ASPs for the treatment of MSSA bacteremia

Variable	No. of studies	No. of patients	OR (95% CI)	*P* Value	I^2^%
Study design					
Retrospective	8	4212	0.71 (0.59–0.84)	0.57	0
Prospective	2	258	0.27 (0.09–0.79)	0.46	0
Location					
USA	5	3670	0.71 (0.59–0.86)	0.52	0
Other	5	800	0.57 (0.36–0.91)	0.25	25.4
Study period					
Initiated before 2008	4	3782	0.58 (0.36–0.92)	0.15	43.4
Initiated after 2008	6	688	0.71 (0.59–0.85)	0.64	0
Mortality recording time					
30-day mortality	6	3676	0.60 (0.48–0.75)	0.81	0
90-day mortality	7	4133	0.69 (0.58–0.82)	0.30	16.9
Adjustment					
Propensity score matched or multiple adjusted	6	4040	0.69 (0.58–0.83)	0.26	23.6
Unadjusted	10	4779	0.74 (0.64–0.87)	0.11	37.6
Control group					
NAF or OXA	7	3910	0.69 (0.57–0.83)	0.26	22.6
CLX	3	560	0.69 (0.42–1.12)	0.46	0

*ASPs* antistaphylococcal penicillins, *CFZ* cefazolin, *CI* confidence interval, *CLX* cloxacillin, *MSSA* methicillin-susceptible *Staphylococcus aureus*, *NAF* nafcillin, *OR* odds ratio, *OXA* oxacillin

### Secondary outcomes

Clinical failure was variably defined and reported in five studies [13, 16, 18, 20, 22]. The cefazolin group had a significantly lower clinical failure (OR, 0.56; 95% CI, 0.37 to 0.85; I^2^ = 44.9%). Eight studies reported the recurrence of bacteremia [13, 15–18, 20–22]. The results indicate no significant difference in the recurrence of bacteremia between the two groups (OR, 1.12; 95% CI, 0.94 to 1.34; I^2^ = 0%). Data on the number of AEs were reported in five studies [15, 16, 18, 20, 22]. No significant differences in AEs rates between the two group were found with high consistency (OR, 0.37; 95% CI, 0.10 to 10.14; I^2^ = 83.1%). The most common AEs included hepatotoxicity (e.g., elevated transaminases), nephrotoxicity (e.g., elevated serum creatinine), and hematotoxicity (e.g., leukopenia, neutropenia). There were no significant differences in the risks of anaphylaxis (OR, 0.91; 95% CI, 0.36 to 2.99; I^2^ = 0%) and hematotoxicity (OR, 0.56; 95% CI, 0.17 to 1.88; I^2^ = 0%). However, we found nephrotoxicity (OR, 0.36; 95% CI, 0.16 to 0.81; I^2^ = 0%) and hepatotoxicity (OR, 0.12; 95% CI, 0.04 to 0.41; I^2^ = 0%) were significantly lower in the cefazolin group than in the ASPs group. Only three studies [13, 16, 22] compared the discontinuation due to AE. The results indicate that cefazolin was associated with lower probability of being discontinuation of treatment due to AEs compared with the ASPs (OR, 0.24; 95% CI, 0.12 to 0.48; I^2^ = 18%) (Table 3 and Additional file 2: Figure S1-S8).

**Table 3:** Meta-analysis of each secondary outcome

Outcome	No. of studies	No. of patients	OR (95% CI)	*P* Value	I^2^%	Effects model
Clinical failure	5	778	0.56 (0.37–0.85)	0.12	44.9	Fixed
Recurrence of bacteremia	8	4017	1.12 (0.94–1.34)	0.80	0	Fixed
AEs	5	672	0.37 (0.10–1.41)	0	83.1	Random
Hepatotoxicity	4	645	0.12 (0.04–0.41)	0.51	0	Fixed
Nephrotoxicity	3	484	0.36 (0.16–0.81)	0.88	0	Fixed
Anaphylaxis	5	672	0.91 (0.36–2.99)	0.53	0	Fixed
Hematotoxicity	4	511	0.56 (0.17–1.88)	0.42	0	Fixed
Discontinuation due to AEs	3	468	0.24 (0.12–0.48)	0.30	18	Fixed

*AEs* adverse effects, *CI* confidence interval, *OR* odds ratio

### Publication bias

**Fig. 3:**
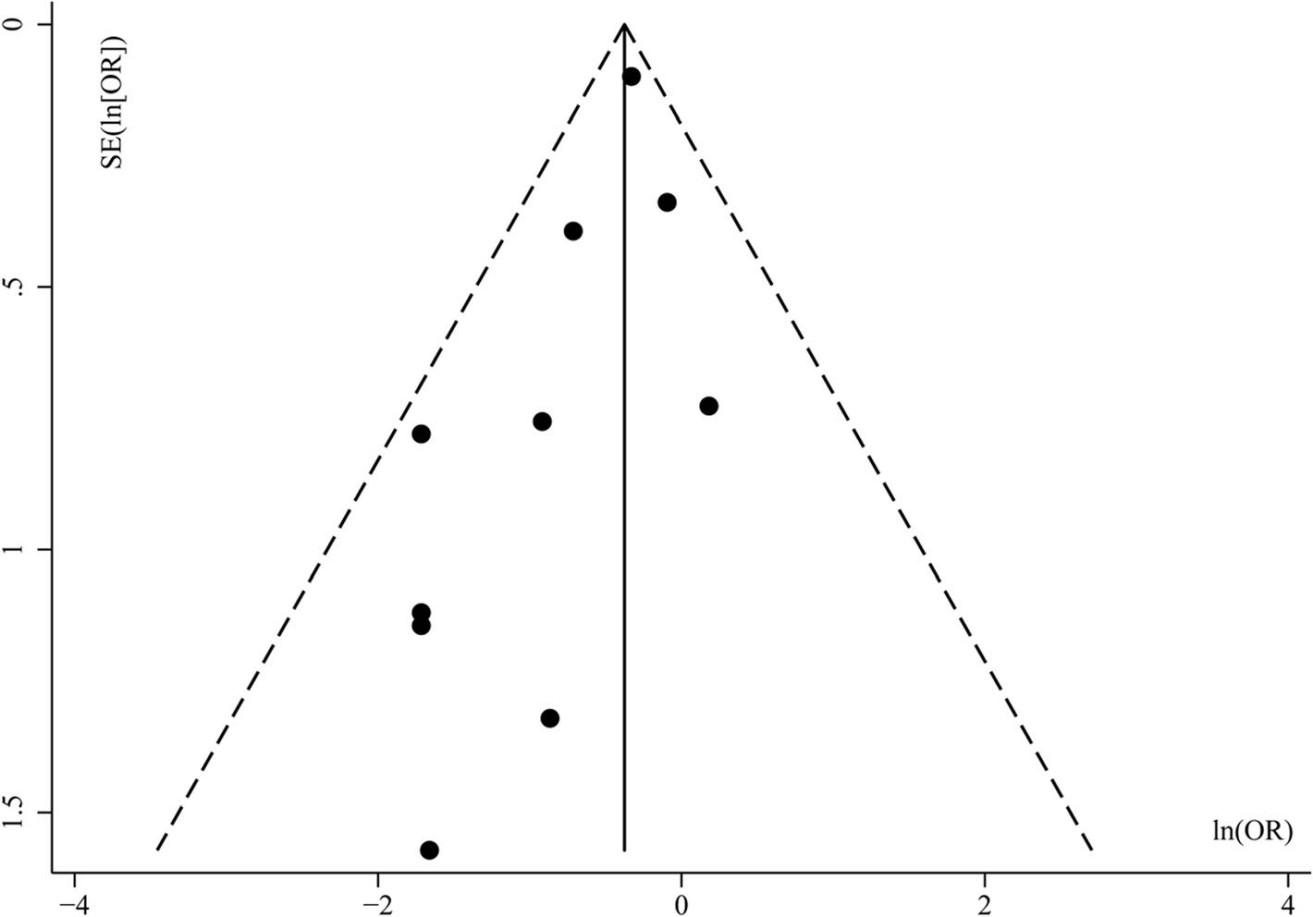
Funnel plots of mortality

Visual inspection of funnel plot showed the presence of a moderate publication bias (Fig. 3). Begg’s test was not significant (*P* = 0.152), but Egger’s test showed the presence of publication bias (*P* = 0.043). Fail-safe methods indicated that 56 publications would be needed to convert our estimated result.

## Discussion

This meta-analysis systematically reviewed studies focusing on the efficacy and safety of cefazolin and ASPs in treating bacteremia caused by MSSA. The results of our meta-analysis demonstrated that cefazolin was associated with a significant reduction in mortality and clinical failure without increasing recurrence of bacteremia, when compared to ASPs. In addition, the safety of cefazolin was superior to ASPs, especially regarding the risk of hepatotoxicity and nephrotoxicity.

The results of the primary outcome were robust and were not significantly altered during most of the subgroup and sensitivity analyses. A subgroup analysis based on the control group showed that the non-significant association in the analysis of the studies in which the control group was cloxacillin could be due to lack of power, as the trend towards lower mortality with cefazolin was evident. Although Egger’s test showed significant publication bias, the fail-safe number was large enough (*n* = 56).

Regarding the safety profile, this present study supported the superior tolerability of cefazolin over ASPs. A recent meta-analysis (published after completion of our work) also reviewed the literature comparing the safety of cefazolin and ASPs for the treatment of MSSA infections [23]. Although the previous meta-analysis focused on MSSA infections but not MSSA bacteremia, these results are consistent with our findings. The results of this study showed that cefazolin was associated with lower rates of nephrotoxicity and hepatotoxicity compared with ASPs among hospitalized patients or outpatients treated for MSSA infections. Moreover, cefazolin was associated with lower probability of discontinuation due to AEs in hospitalized patients and hypersensitivity reactions in outpatients [23].

In clinical practice, the preference for ASPs over cefazolin for MSSA bacteremia is primarily due to concerns about the inoculum effect. The inoculum effect has been defined as a significant elevation in the cefazolin minimum inhibitory concentration with an inoculum higher than standard bacterial inoculum [24]. This is usually due to the production of a type A β-lactamase that can hydrolyze cefazolin [25]. Two recent review articles summarized clinical reports focused on cefazolin inoculum effect [9, 10]. However, most of these reports are case reports or case series and no study show any significant difference in outcomes when comparing isolates with or without the inoculum effect [26–28]. A recent study by Lee et al. found that treatment failure (61.5% vs. 28.9%) and mortality (15.4% vs. 0%) were significantly higher in the inoculum effect-positive arm than in the negative arm among patients who received cefazolin [22]. Therefore, the clinical relevance of the cefazolin inoculum effect is still unclear. More properly designed studies comparing cefazolin to ASPs for high-inoculum MSSA bacteremia, such as endocarditis, are required.

The selection of a regimen for the treatment of MSSA bacteremia is primarily based on efficacy, safety, costs and availability. In addition to the reduction in mortality, clinical failure, risks of hepatotoxicity and nephrotoxicity, and the probability of discontinuation due to AEs as noted above, cefazolin offers several additional advantages such as being less expensive and being more convenient to dose [15, 18]. Taken together, the evidence of supports a beneficial effect of cefazolin over ASPs for MSSA bacteremia in the absence of endocarditis or high-burden infection.

Two recent meta-analyses (both published after completion of our work) have compared the use of cefazolin and ASPs for the treatment of MSSA bacteremia [29, 30]. The conclusions of these two meta-analyses were similar and consistent: the survival rates and safety profiles of cefazolin were superior to ASPs. The meta-analysis conducted by Bidell et al. concluded that cefazolin was associated with a significant reduction in all-cause 90-day mortality (OR, 0.63; 95% CI, 0.41 to 0.99; I^2^ = 58%) and discontinuation due to AEs (OR, 0.25; 95% CI, 0.11 to 0.56; I^2^ = 13%), when compared to ASPs [29]. Another meta-analysis conducted by Rindone et al. demonstrated that the mortality (RR, 0.78; 95% CI, 0.69–0.88; I^2^ = 51%) and withdrawals from AEs (RR, 0.27; 95% CI, 0.16 to 0.47; I^2^ = 37%) were significantly lower in the cefazolin group than in the ASPs group [30]. These two meta-analyses had some differences compared with ours: (i) these two previous meta-analyses did not report the differences in AEs rates. Our study compared the differences in AEs (e.g., hepatotoxicity, nephrotoxicity, hematotoxicity, and anaphylaxis) between the cefazolin and ASPs and concluded the safety of cefazolin was superior to ASPs, especially regarding the risk of hepatotoxicity and nephrotoxicity; (ii) our meta-analysis demonstrated that the clinical failure was significantly lower in the cefazolin group than in the ASPs group. However, the meta-analysis by Bidell et al. found no significant differences in clinical failure rate between the two groups [29]. The difference may be due to the included studies. Of note, the meta-analysis conducted by Bidell et al. included only seven out of the ten observational studies analyzed in our meta-analysis. Moreover, two original studies [17, 21] included in the meta-analysis did not report the clinical failure rate. However, these two original studies were used in the analysis of clinical failure in the meta-analysis conducted by Bidell et al. [29]; (iii) all analyses were conducted unadjusted in these two previous meta-analyses. It is commonly believed that adjusted estimates are consistently closer to the true values than unadjusted estimates. The present meta-analysis used the adjusted estimates if appropriate and presented separate subgroup analyses of adjusted or unadjusted estimates.

There were several limitations that should be considered. First, all studies included in the present meta-analysis were observational studies, which have a high selection bias and may expose the analysis to confounders. For example, patients in the ASPs treatment arms had numerically higher rates of endocarditis [13, 17, 18, 20, 22] and ICU admission [16, 17, 19, 21]. The subgroup analysis of the adjusted odds ratios of primary outcome continued to favor cefazolin. Although these publications tried to adjust for the confounders, residual confounding factors remain. Second, despite the absence of statistical heterogeneity, the inherent heterogeneity across the studies analyzed (i.e., source of bacteremia, source control, dose and duration, etc.) should not be discounted. Third, as the source of bloodstream infection were diverse in the present study, we could not conclude that cefazolin was superior to ASPs for a specific type of bacteremia, especially in deep-seated infection, such as endocarditis.

## Conclusion

The results of present study favor the application of cefazolin for the treatment of MSSA bacteremia. Therefore, we suggest that cefazolin be used as a first-line regimen for MMSA bacteremia in the absence of endocarditis or high-burden infection. Our study should be regarded as important evidence to help make clinical decisions in choosing a treatment option for MSSA bacteremia.

## Additional files

Additional file 1: **Table S1.** Characteristics of the studies included in the meta-analysis. **Table S2.** Quality of assessment for included studies. **Table S3.** Sensitivity analysis assessing mortality. (DOCX 24 kb)

Additional file 2: Forest plots of odds ratios for second outcomes (see in **Figure S1-S8**). (DOCX 11351 kb)

## Abbreviations

AEs: Adverse effects
ASPs: Antistaphylococcal penicillins
CFZ: Cefazolin
CIs: Confidence intervals
CLX: Cloxacillin
MC: Multicenter
MRSA: Methicillin-resistant *Staphylococcus aureus*
MSSA: Methicillin-susceptible *Staphylococcus aureus*
NAF: Nafcillin
NOS: Newcastle-Ottawa scale
NR: Not reported
OR: Odds ratio
OXA: Oxacillin
PS: Propensity score
SC: Single center
