# Supplementary material for: Efficacy and safety of cefazolin versus antistaphylococcal penicillins for the treatment of methicillin-susceptible Staphylococcus aureus bacteremia: a systematic review and meta-analysis
Source: BMC Infect Dis. 2018 Oct 11;18:508. doi: 10.1186/s12879-018-3418-9 (PMC6180622; doi:10.1186/s12879-018-3418-9)
Supplement: Supplementary file 1 — Table S1. Characteristics of the studies included in the meta-analysis. Table S2. Quality of assessment for included studies. Table S3. Sensitivity analysis assessing mortality. (DOCX 24 kb) [file 12879_2018_3418_MOESM1_ESM.docx]

**Table S1** Characteristics of the studies included in the meta-analysis

| Study | Treatment group | Type of bacteremia (%) | | | | | | | | | Source of bacteremia acquisition % | Metastatic infection % | Source control % |
| --- | --- | --- | --- | --- | --- | --- | --- | --- | --- | --- | --- | --- | --- |
|  |  | Primary | Pneumonia | Endocarditis | CR | Bone and joint | SSTI | UTI | CNS | Other |  |  |  |
| Lee 2011 | CFZ | 27 | 8 | 2 | 22 | NR | 20 | NR | NR | NR | CA:39 | 16 | 29 |
|  | NAF | 23 | 13 | 16 | 20 | NR | 12 | NR | NR | NR | CA:51 | 27 | 26 |
| Paul 2011 | CFZ | 22.6 | 10 | 6.5 | 11.6 | 5 | 15.3 | NR | NR | 12.2 | CA: 12.2, HCA: 38.3, HA: 49.5 | NR | NR |
|  | CLX |  |  |  |  |  |  |  |  |  |  |  |  |
| Renaud 2011 | CFZ | 14.3 | NR | NR | 35.7 | NR | NR | NR | NR | NR | NR | NR | NR |
|  | CLX | 23.1 | NR | NR | 38.5 | NR | NR | NR | NR | NR | NR | NR | NR |
| Li 2014 | CFZ | 14 | 4 | 25 | 10 | 31 | 14 | 3 | NR | NR | NR | 34 | 67.8 |
|  | OXA | 6 | 6 | 12 | 3 | 59 | 3 | 12 | NR | NR | NR | 35 | 61.8 |
| Bai 2015 | CFZ | 33 | 16 | 2 | 12 | 14 | 25 | 6 | NR | 7 | CA: 26, HCA: 39, HA: 35 | NR | 63.3 |
|  | CLX | 34 | 18 | 12 | 14 | 11 | 17 | 7 | NR | 14 | CA: 37, HCA: 31, HA: 32 | NR | 58 |
| Rao 2015 | CFZ | NR | 1.9 | 16.5 | 45.6 | 20.4 | 14.6 | 1.9 | 0 | NR | NR | 29.1 | 76.7 |
|  | OXA | NR | 1.7 | 20.7 | 24.1 | 13.8 | 22.4 | 1.7 | 1.7 | NR | NR | 19 | 51.7 |
| Pollett 2016 | CFZ | 40 | 1.4 | 14 | 19 | 7.1 | NR | 3 | NR | 4 | HA: 11 | NR | NR |
|  | NAF | 53 | 13.3 | 17 | 10 | 10 | NR | 3 | NR | 0 | HA: 23 | NR | NR |
| Flynt 2017 | CFZ | NR | NR | 16.2 | NR | 19.1 | 23.5 | 5.9 | NR | 8.8 | NR | NR | NR |
|  | NAF | NR | NR | 27.2 | NR | 24.7 | 19.8 | 8.7 | NR | 13.6 | NR | NR | NR |
| McDanel 2017 | CFZ | NR | NR | 4 | NR | NR | 25 | NR | NR | NR | HA: 21 | NR | NR |
|  | NAF | NR | NR | 7 | NR | NR | 23 | NR | NR | NR | HA: 24 | NR | NR |
| Lee 2018 | CFZ | 15.2 | 3.8 | 1.3 | 12.7 | 35.4 | 34.2 | 0 | 0 | 1.3 | HCA: 27.7 | 12.7 | NR |
|  | NAF/OXA | 13.5 | 9.8 | 6.7 | 12.3 | 37.4 | 23.3 | 2.9 | 0.6 | 1.8 | HCA: 2.8 | 14.1 | NR |

CA, community-acquired; CFZ, cefazolin; CLX, cloxacillin; CNS, central nervous system; CR, catheter-related; HA, hospital acquired; HCA, healthcare-associated; NAF, nafcillin; NR, not reported; OXA, oxacillin; SSTI, skin and soft tissue infection; UTI, urinary tract infection.

**Table S2** Quality of assessment for included studies

| Study | Total (0-9) | Selection (0-3) | Comparability (0-2) | Exposure/ Outcome (0-4) |
| --- | --- | --- | --- | --- |
| Lee 2011 | 8 | 3 | 2 | 3 |
| Paul 2011 | 7 | 3 | 2 | 2 |
| Renaud 2011 | 4 | 2 | 1 | 1 |
| Li 2014 | 6 | 3 | 0 | 3 |
| Bai 2015 | 7 | 3 | 2 | 2 |
| Rao 2015 | 5 | 2 | 2 | 1 |
| Pollett 2016 | 9 | 4 | 2 | 3 |
| Flynt 2017 | 5 | 2 | 2 | 1 |
| McDanel 2017 | 7 | 3 | 2 | 2 |
| Lee 2018 | 8 | 4 | 2 | 2 |

**Table S3** Sensitivity analysis assessing mortality

| Study Omitted | OR (95% Confidence Interval) | P Value | I^2^ % |
| --- | --- | --- | --- |
| Lee 2011 | 0.69 (0.58-0.83) | 0.45 | 0 |
| Paul 2011 | 0.67 (0.56-0.81) | 0.38 | 6.7 |
| Renaud 2011 | 0.69 (0.58-0.82) | 0.33 | 12.8 |
| Li 2014 | 0.69 (0.58-0.82) | 0.37 | 7.5 |
| Bai 2015 | 0.70 (0.58-0.84) | 0.58 | 6.3 |
| Rao 2015 | 0.69 (0.58-0.83) | 0.44 | 0 |
| Pollett 2016 | 0.69 (0.58-0.83) | 0.36 | 9.1 |
| Flynt 2017 | 0.68 (0.57-0.81) | 0.37 | 8.3 |
| McDanel 2017 | 0.56 (0.37-0.84) | 0.42 | 1.6 |
| Lee 2018 | 0.70 (0.59-0.83) | 0.61 | 0 |
