# Supplementary material for: Efficacy and safety of cefazolin versus antistaphylococcal penicillins for the treatment of methicillin-susceptible Staphylococcus aureus bacteremia: a systematic review and meta-analysis
Source: BMC Infect Dis. 2018 Oct 11;18:508. doi: 10.1186/s12879-018-3418-9 (PMC6180622; doi:10.1186/s12879-018-3418-9)
Supplement: Supplementary file 2 — Forest plots of odds ratios for second outcomes (see in Figure S1-S8). (DOCX 11351 kb) [file 12879_2018_3418_MOESM2_ESM.docx]

**
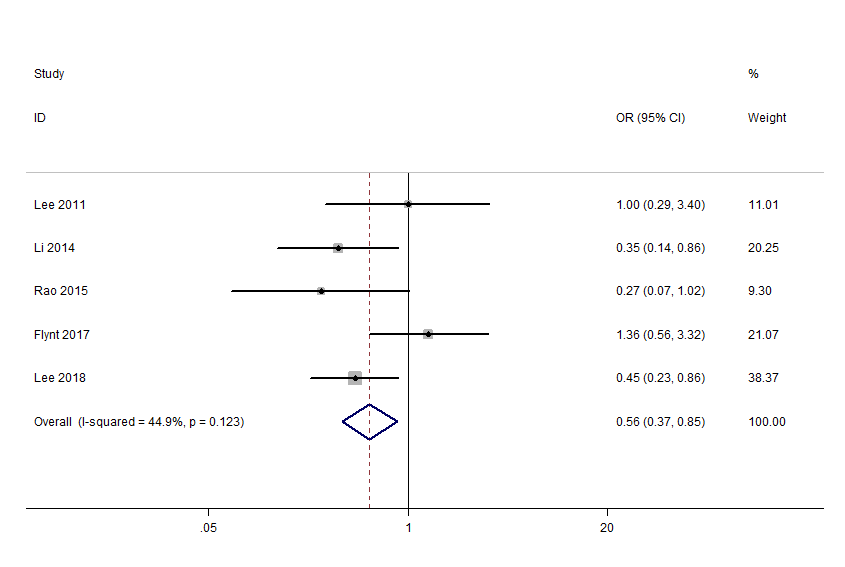
Figure S1** Forest plots of odds ratios for clinical failure


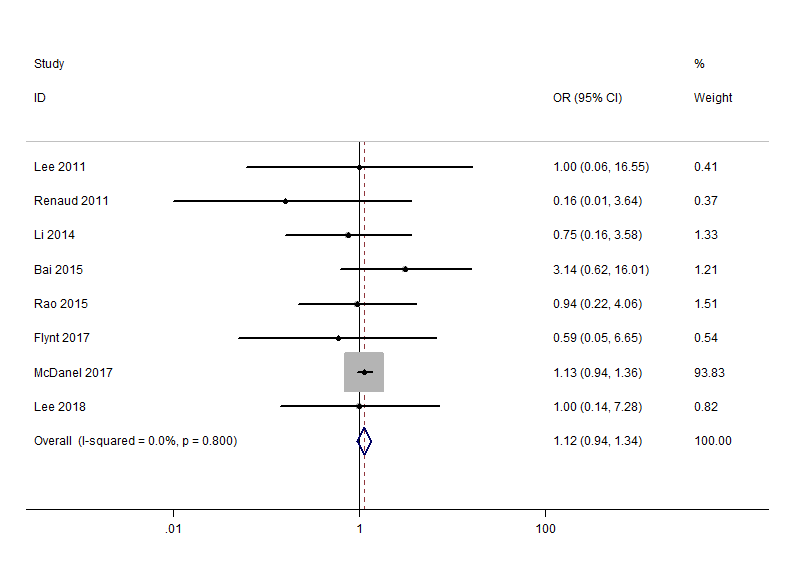


**Figure S2** Forest plots of odds ratios for recurrence of bacteremia


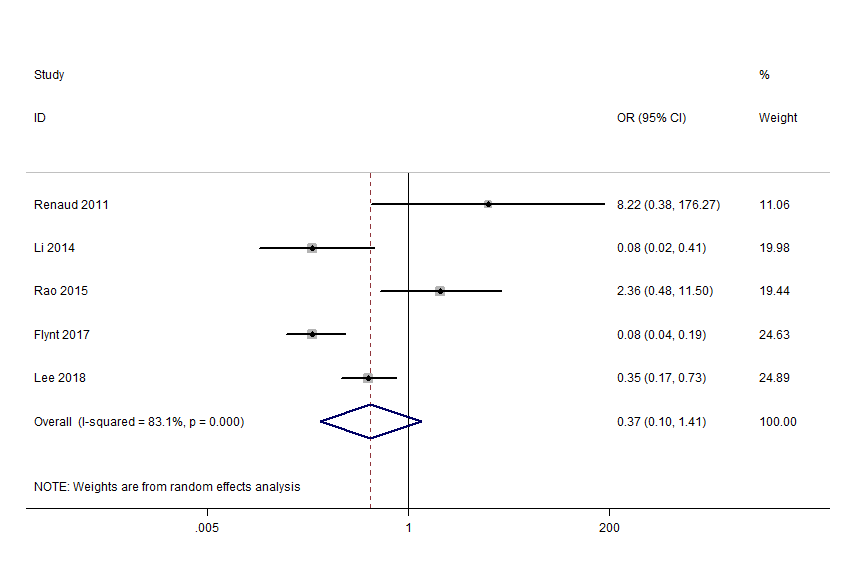


**Figure S3** Forest plots of odds ratios for adverse effects rates


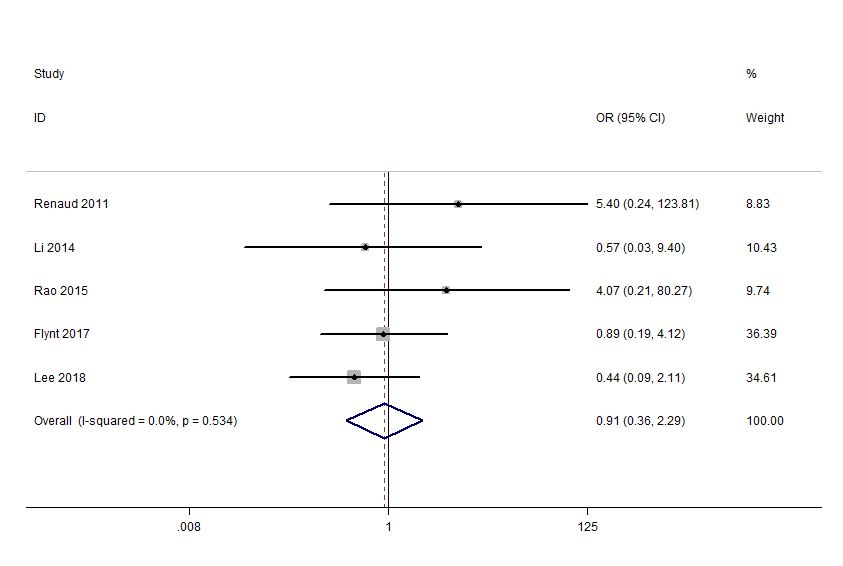


**Figure S4** Forest plots of odds ratios for anaphylaxis


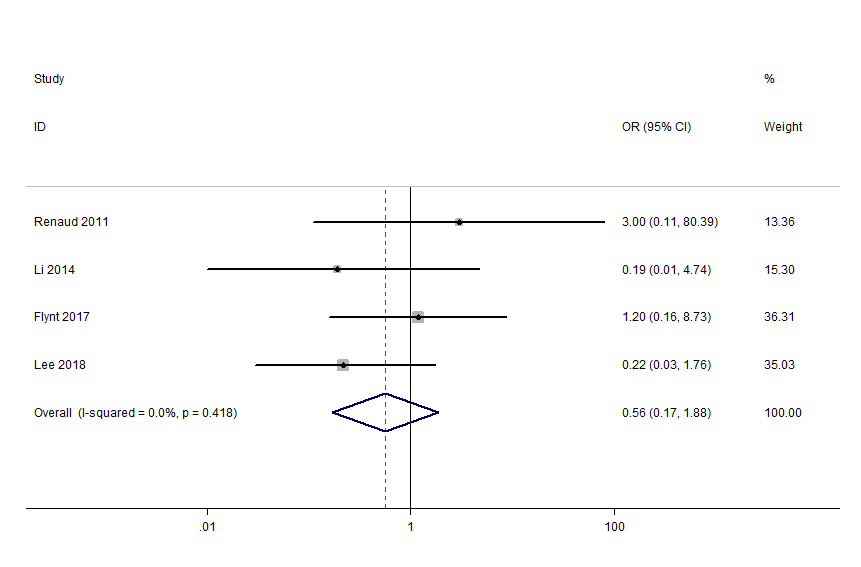
**Figure S5** Forest plots of odds ratios for hematotoxicity


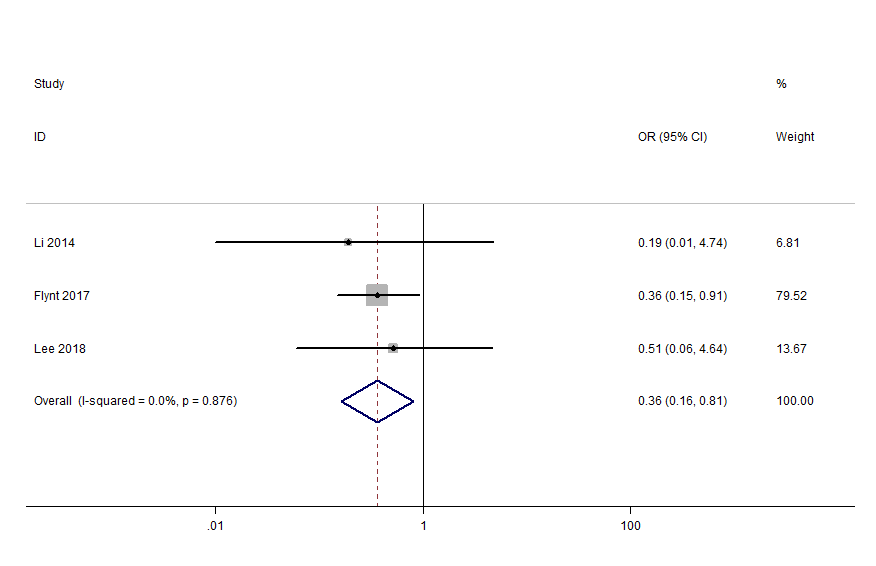


**Figure S6** Forest plots of odds ratios for nephrotoxicity


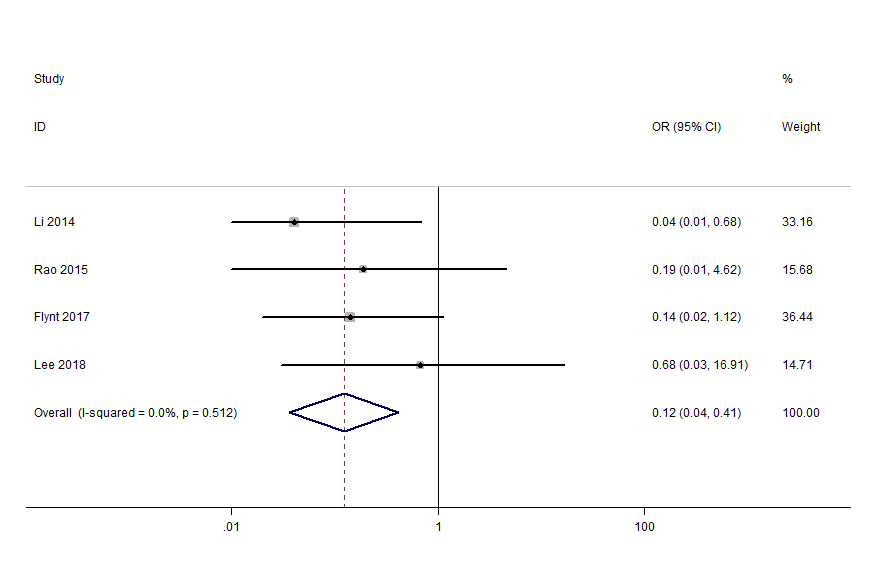


**Figure S7** Forest plots of odds ratios for hepatotoxicity


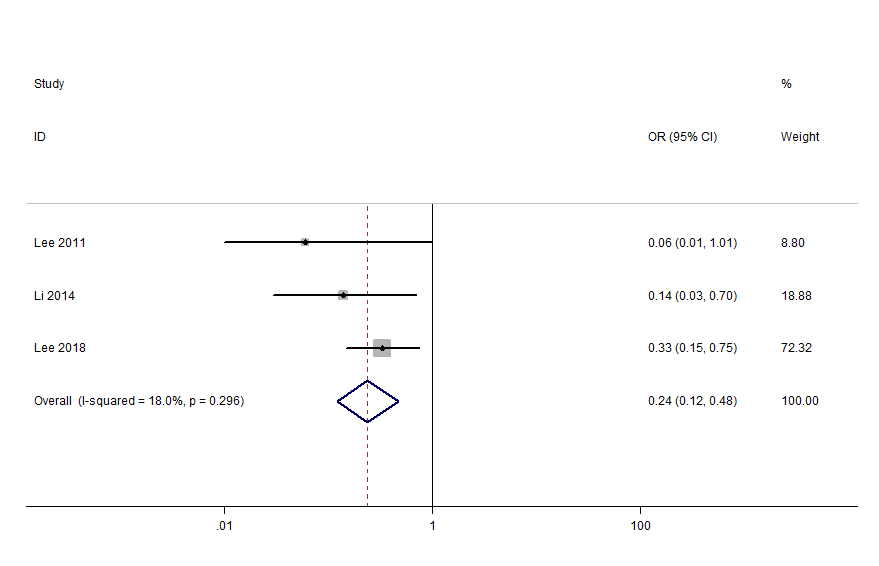


**Figure S8** Forest plots of odds ratios for discontinuation of treatment due to adverse effects
